# An assessment of the Henssge method for forensic death time estimation in the early post-mortem interval

**DOI:** 10.1007/s00414-024-03338-5

**Published:** 2024-09-24

**Authors:** Fabian Heinrich, Felix Rimkus-Ebeling, Eric Dietz, Tobias Raupach, Benjamin Ondruschka, Sven Anders-Lohner

**Affiliations:** 1 Institute of Legal Medicine, University Medical Center Hamburg-Eppendorf, Hamburg, Germany; 2 Department of Medical Statistics, London School of Hygiene and Tropical Medicine, London, UK; 3 Centre for Data and Statistical Science for Health, London School of Hygiene and Tropical Medicine, London, UK; 4 Institute for Medical Education, University Hospital Bonn, Bonn, Germany

**Keywords:** Forensic science, Time-since-death, Henssge method, Body mass index, Body surface area, Ambient temperature, Post-mortem interval, Time of death

## Abstract

**Background:**

Time-since-death (TSD) diagnostics are crucial in forensic medical casework. The compound method by Henssge and Madea, which combines temperature and non-temperature-based techniques, is widely used to estimate TSD. This study aims to validate the predictive ability of this method in a cohort of 76 deceased individuals with known times of death (TOD).

**Methods:**

A convenience sample of 76 deceased individuals was examined at the Institute of Legal Medicine, University Medical Center Hamburg-Eppendorf. The study included individuals who died at the hospital and those with sudden death in public. Exclusion criteria included age under 18, known infection or sepsis, polytrauma, bleeding, and hyperthermia. The TSD interval was calculated using the Deathtime software.

**Results:**

The overall agreement between the actual TOD and the 95% prediction interval for the TSD was 36.8% (95% CI: 26.1 to 48.7). Warm-stored corpses showed a higher agreement (61.9% [95% CI: 38.4 to 81.9]) compared to cold-stored corpses (27.3% [95% CI: 16.1 to 41.0]). Factors such as body mass index (BMI) and body surface area (BSA) were found to influence the odds of agreement. Assuming a plausible range of ambient temperatures between death and admission improved the agreement in cold-stored cases.

**Conclusion:**

The study found low to moderate agreement between the actual TOD and the 95% prediction interval using the Henssge method. Incorporating BMI and BSA could improve the predictive accuracy of TSD estimations. Further research with larger sample sizes and external validation is recommended to refine the model.

**Supplementary Information:**

The online version contains supplementary material available at 10.1007/s00414-024-03338-5.

## Introduction

Time-since-death (TSD) diagnostics is fundamental to forensic medical casework. In the early post-mortem interval, the compound method, according to Henssge and Madea, has been established on an international scale, which combines temperature and non-temperature-based examination techniques to determine the TSD and can be carried out directly at the scene of the crime or the place of discovery of a body. The method estimates the TSD interval, considering the rectal temperature, ambient temperature, body weight, clothing or covering, and environmental conditions [1]. The temperature-based method is supplemented by non-temperature-based methods that can further delimit the TSD interval. These include the formation of rigor and livor mortis, blanchability and shifting of livor mortis, the electrical excitability of the mimic musculature, the re-establishment of rigor mortis after mechanical dissolving, the triggering of an idiomuscular bulge by mechanical stimulation of the skeletal musculature as well as the pharmacological excitability of the pupillomotor system [2, 3]. The non-temperature-based methods are based on upper- and lower-time limits of observed triggerability, partly supported by sparse literature with limited empirical data. For instance, it was shown that the upper time limit of the re-establishment of rigor mortis after mechanical dissolving requires re-evaluation [4, 5] and that one should refrain from applying methods for pharmacological excitability of pupillomotor function by atropine and tropicamide to forensic TSD diagnostics [6]. In contrast, the upper time limit for triggering an idiomuscular contraction through mechanical stimulation of skeletal muscles was previously based on a single case report, and more extensive experiments have recently confirmed the time limit [7].

The model of the temperature-based estimation of TSD is based on the monocentric work of Henssge [8–11], who carried out post-mortem temperature measurements on 39 deceased persons with a known time of death [8]. The study included corpses with a known time of death (TOD) without imposing further restrictions on the study sample, meaning individuals with varying ages (range: [0.9, 93] years) and weights (range: [9,112] kg) were included. Times and conditions from death until the first measurement were neglected, with controlled storage conditions and first measurements established within 1 to 6 h after death. Henssge’s equations adjust for clothing, environmental conditions (using a singular corrective factor [CF]), and body weight (W) using a multiplicative scaling factor between the correction factor and weight. Some potential predictors were not included in the equations without further justification. Nevertheless, body surface and adipose insulation likely impact the cooling kinetics of deceased individuals [12]. Last, the model is prone to overfitting and lacks transportability with equations established on the full dataset without internal or external validation and without accuracy information. Nomogram correction factors were later derived based on two cohorts of 53 deceased under standard conditions and 26 deceased under varying cooling conditions [13].

So far, only a few case series and field studies have been published examining the predictive ability of the temperature and compound method [12, 14–19]. Primarily, these focus on temperature-based TSD diagnostics. Some of these studies compared the results of forensic death time estimation to the probable TSD interval narrowed down by police investigations, as the actual time of death (TOD) was unknown. In such a scenario, given the uncertainty in the TSD interval narrowed down by police investigations, even when there was an overlap between the 95% prediction interval and the TSD interval narrowed by police investigations, the actual TOD may not be within the 95% prediction interval from the Henssge equation. Conversely, a lack of overlap between the intervals does not mean it is incompatible.

The present work aims to examine the predictive ability of the temperature and compound method according to Henssge [8–11] in a cohort of 76 deceased individuals with a known TOD to investigate the effect of factors that presumably influence the cooling kinetics of corpses on the odds of compatibility between the actual TOD and the 95% prediction interval for the TSD and examine the cohort-specific effect of accounting for a plausible range of ambient temperatures during the unobserved interval from death until admission.

## Material and methods

### Study inclusion and exclusion criteria

The examinations were performed on a convenience sample of 76 deceased individuals with a known time point of death who were admitted to the Institute of Legal Medicine (ILM) of the University Medical Center Hamburg-Eppendorf. The study population consisted of individuals who had died at the University Medical Center (*n* = 53) and individuals who died in public after observed sudden death and unsuccessful out-of-hospital resuscitation (*n* = 23). In the latter case, the end of resuscitation measures was considered the TOD. Only a maximum uncertainty of 30 min regarding TOD was accepted for inclusion in the study.

The exclusion criteria were age under 18, a previously known infection or sepsis, polytrauma, bleeding, and hyperthermia (rectal temperature at admission to the ILM > 37 °C). There were no signs of putrefaction or suspicion of intoxication in any of the cases. The exact cause of death remained unknown in most cases, with conventional autopsies conducted in a minority of cases.

### Study enrolment procedure

Enrolment in the study was conducted by an investigator (ED) who did not perform postmortem examinations or calculate the postmortem interval with knowledge of the TOD. This investigator also determined when the tests were conducted, between 8 and 20 h postmortem. The examinations on the deceased were carried out by another examiner (FR-E, in some cases, in cooperation with SA-L) without knowledge of the TOD. Calculation of the 95% prediction interval for the TSD interval was also carried out without knowing the TOD (SA-L, FR-E). Information on the TOD was unblinded during data analysis.

All deceased were placed on a metal stretcher on a cotton sheet and were unclothed from the time of admission to the Institute of Legal Medicine. Until the examinations, the deceased were stored at a constant room temperature of about 20 °C (*n* = 21) or about 10 °C (*n* = 55; large cold-store room) in still air. Assignment to groups with different storage temperatures did not take place at random. Where corpses were already stored in the cooling chamber by the forensic assistants, they were included as cold stored. There is no direct or indirect association with the exposure or outcome; thus, no strong systematic bias is expected.

### Study examination procedures

At the time point defined for the examinations (ED), the measurements were performed as described below, analogous to the methodology commonly used in forensic casework [1, 2].

Rectal temperature was measured at a depth of at least 10 cm using a calibrated thermometer with a suitable probe (Testo 110, Testo, Germany). Using the same thermometer, after connecting a probe to measure the air temperature, the ambient temperature was measured where the deceased was stored in the institute.

The degree of rigor mortis was recorded semiquantitatively. The degree was classified according to the degree of expression at the joints of the extremities (elbow, knee). Mild muscular resistance that the examiner could easily overcome through repeated joint movement was classified as moderate. Marked near-maximal joint stiffness was classified as strong. Cases with marked rigor mortis that did not meet the criteria for classification as moderate or strong were classified as intermediate.

Rigor mortis was entirely released by repeated movement of the elbow joints. The reformation of rigor mortis was tested manually 2 h later, according to the practical requirements of forensic casework. The intensity of reformation was recorded semiquantitatively (negative, moderate, intermediate, strong) and was compared with the intensity before the examination. The reformation was recorded as positive if it was graded as moderate, medium, or strong and negative if no renewed increase in muscle resistance was noted after release. Graduation was also graded positive in the case of unilateral reformation.

Livor mortis was classified as moderate or intense by visual inspection. After vigorous thumb pressure in the central area of livor mortis, the degree of fading produced was graduated as negative, incomplete, or complete semiquantitatively.

The mechanical excitability of skeletal muscle (idiomuscular contraction) was tested bilaterally on the muscle belly of the biceps brachii muscle by a single, forceful blow with a rounded metal rod. The local muscular response was recorded visually and by palpation after 20 s and graduated as negative or positive. Graduation as positive was also done in the case of unilateral triggering.

Electrical excitability of the mimic musculature was tested using insertion electrodes and a stimulation device (MD 95/2007, 40 V, Funeralia, Germany) with the placement of the electrodes in the upper eyelid and classified as negative or positive (grade 1 to 6) according to [2].

Due to previous findings suggesting that this method is not suitable for forensic TSD diagnosis [6], postmortem pharmacological susceptibility of pupillomotor function was not included in the methodology of this study. In addition, examinations of the shifting of livor mortis were not performed to not influence the appearance of the deceased during the examinations.

### Calculations of the TSD

The TSD interval was calculated using the Deathtime software (AMA Soft, Germany; www.amasoft.de). Since the program sets the time limit for the re-formation of rigor mortis after mechanical release at 9.5 h post-mortem (hpm), this was manually corrected to 20 hpm according to recent results, if relevant [4, 5]. A correction factor 1.0 was used for body weight based on the deceased's storage conditions.

For each of the 76 cases, the following three calculations were made (a-c):

#### Assuming a constant ambient temperature from death until measurement – compound method

The ambient temperature measured at the deceased's storage site at the Institute (${T}_{amb}$) was assumed for the time from death until measurement; non-temperature-based methods were included (i.e., neglecting potentially different ambient temperatures before admission according to Henssge [8]).

#### Taking a plausible range of ambient temperatures from death until admission into account – temperature-based methods only

Due to the neglect of times and storage conditions before admission, a corrected mean ambient temperature was used for calculation $\left({T}_{corr}\right)$. An exploratory approach assumed a range of plausible indoor ambient temperatures (${{\boldsymbol{T}}}_{am{b}^{*}}$, a vector of length 11) between 19 and 29 °C from death until admission. The temperature range was chosen considering a clothing and covering state of the deceased; non-temperature-based methods were neglected. A weighted average was calculated as shown in Formula 1.

Formula 1. The formula for calculating the corrected ambient temperature $\left({T}_{corr}\right)$ as the weighted average of a vector with a plausible range of ambient temperatures before admission $\left({T}_{am{b}^{*}}\right)$ and the storage temperature (${T}_{amb}$) at the ILM.

$${T}_{corr}= \frac{\left({t}_{prae}*{{\boldsymbol{T}}}_{am{b}^{*}}+\left({t}_{hpm}-{t}_{prae}\right)*{T}_{amb}\right)}{{t}_{hpm}}$$

, where t_prae_ is the time from death until admission at the ILM [hours] and t_hpm_ is the time from death until measurements [hours].

#### Taking a plausible range of ambient temperatures from death until admission into account – compound method

Due to the neglect of times and storage conditions before admission, a corrected mean ambient temperature was used for calculation $\left({T}_{corr}\right)$. In part (b), an ambient temperature that optimised compatibility in the actual TOD and the 95% prediction interval for this cohort was obtained. This cohort-specific ambient temperature was used $\left({T}_{am{b}^{*}}\right)$; non-temperature-based methods were considered. A weighted average was calculated as shown in Formula 1.

### Exposure

The primary exposure was time from death until measurement.

### Outcome

The primary outcome was compatibility between the actual TOD and the 95% prediction interval for the TSD, according to Henssge. Compatibility was defined as an actual TOD within the 95% prediction interval for the TSD.

### Collection and recording of potential confounding variables

Confounder variables were defined on a clinical basis. Sex [binary], age [continuous], body mass index [in kg/m^2^, continuous], body surface area (m^2^, continuous [20]) time from death until arrival at the ILM [in hours, continuous], and storage time from admission to the ILM until examination [in hours, continuous] were recorded by FR-E and SA-L.

### Descriptive statistics

Categorical variables were summarised with numbers and percentages, and continuous variables were summarised using mean and SD or median and IQR, as appropriate. Continuous variables were inspected for approximate normality using Q-Q plots and histograms.

### Comparative statistics

Tables were used to compare two categorical variables, and the ${\chi }^{2}$ test was used to formally assess the null hypothesis that there was no difference in expected and observed values across groups. Summary statistics and boxplots were used to compare continuous variables across two groups. A Mann–Whitney U test was conducted to compare locations of continuous variables between two groups where substantial violations of the normality assumption were observed, and a location shift was plausible.

Exact binomial 95% confidence intervals were calculated for proportions.

### Generalised linear model

Generalised linear models were used to investigate the association between the (i) time from death until admission, (ii) time from admission until measurement, (iii) body mass index, (iv) body surface area and (v) mode of storage, and the odds of compatibility between the actual TOD and the 95% prediction interval for the TSD. A logistic regression was used. Agreement in the actual TOD and the 95% prediction interval for the TSD, defined as compatibility of the actual TOD and the 95% prediction interval for the TSD, was used as the dependent variable in the model [binary]. First, we calculated univariable logistic regressions by including each interest factor separately. Then, we fitted multivariable logistic regression models by including the factor of interest and confounding variables for each exposure-outcome relationship. Confounders were a priori-defined on a clinical basis, as shown in the directed acyclic graphs (DAGs, Supplementary Figs. 1–4). Likewise, interaction terms were included on a clinical basis. The analysis involved testing for misspecification of the linear predictor using the Hosmer–Lemeshow test with ten groups and predicting standardised Pearson residuals. Index plots and scatter plots were used to identify influential values and assess the correct functional form of continuous variables.

Empirical standardisation was used to calculate marginal probabilities. The delta method was used to estimate 95% confidence intervals for marginal probabilities.

The dataset was inspected for missing data using tabulations as appropriate. Missing data proportions yielded below 5% for all variables, and a complete case analysis was employed. Generally, only one missing value was observed for the re-establishment of rigor mortis (*n* = 1). One case had to be excluded from calculation (b) at assumed ambient temperatures of 26 °C to 29 °C because the body and ambient temperatures were too close to each other to allow determination of the TSD interval (*n* = 1).

Statistical analyses were performed using STATA/MP 18.0 (StataCorp, Texas, USA). The figures were created using Adobe Illustrator (Adobe Inc, CA, USA) and GraphPad Prism (GraphPad Software, MA, USA). Tests were conducted at a 5% significance level.

## Results

Of the 76 cases studied, 30 were female (39.5%) and 46 were male (60.5%). The median age was 73.0 years (IQR: 63 to 78), the median BMI was 25.5 kg/m^2^ (IQR: 21 to 31), and the median BSA was 1.9 m^2^ (IQR: 1.7 to 2.1). The median time from death until admission to the ILM was 4.5 h (IQR: 3 to 7). The median time from admission until measurement was 7.5 h (IQR: 5 to 9).

The median baseline ambient temperature of the 21 bodies stored in warmer conditions at the institute was 19.7 °C (IQR: 19 to 20), and the median ambient temperature of the cases stored in cooler conditions was 9.1 °C (IQR: 9 to 10). Descriptive statistics stratified by the storage conditions in the institute are shown in Table 1.

**Table 1:** Descriptive statistics of baseline statistics of individuals in the study stratified by the storage conditions

	Cold storage Median (IQR)^1^ and numbers (%)	Warm storage Median (IQR)^1^ and numbers (%)	P-value	Total Median (IQR)^1^ and numbers (%)
	*N* = 55	*N *= 21		*N* = 76
Age, years	74.0 (64.0–80.0)	69.0 (54.0–76.0)	0.07	73.0 (63.0–78.0)
Sex			0.88	
Female	22 (40.0%)	8 (38.1%)		30 (39.5%)
Male	33 (60.0%)	13 (61.9%)		46 (60.5%)
BMI, kg/m^2^	24.2 (21.2–30.0)	28.4 (23.6–30.8)	0.15	25.5 (21.4–30.6)
Rectal temperature, °C	29.1 (26.7–32.2)	30.4 (29.1–32.0)	0.06	29.8 (27.4–32.1)
Time from death until admission, hours	4.8 (3.5–7.2)	3.5 (1.5–4.5)	0.004	4.5 (3.2–6.8)
Time from admission until measurement, hours	7.0 (4.8–9.2)	8.0 (6.8–9.2)	0.30	7.5 (5.4–9.2)
Body surface area, m^2^	1.9 (1.6–2.1)	2.0 (1.9–2.1)	0.16	1.9 (1.7–2.1)

Abbreviations: *BMI* body mass index, *IQR* interquartile range

### (a) Assuming a constant ambient temperature from death until measurement – compound method according to Henssge

When the storage temperature of the deceased after admission to the ILM was used to calculate the TSD interval (calculation a), the actual TOD was compatible with the estimated 95% prediction interval for the TSD in 28 cases (36.8% [95% CI: 26.1 to 48.7]). In contrast, the actual TOD lay outside the calculated 95% prediction interval for the TSD in 48 cases (63.2% [95% CI: 51.3 to 73.9]). Among 95% prediction intervals not compatible with the actual TOD, 19% of the 95% prediction intervals overestimated the actual TOD (n = 9) and 81% underestimated the actual TOD (*n* = 39).

**Fig. 1:**
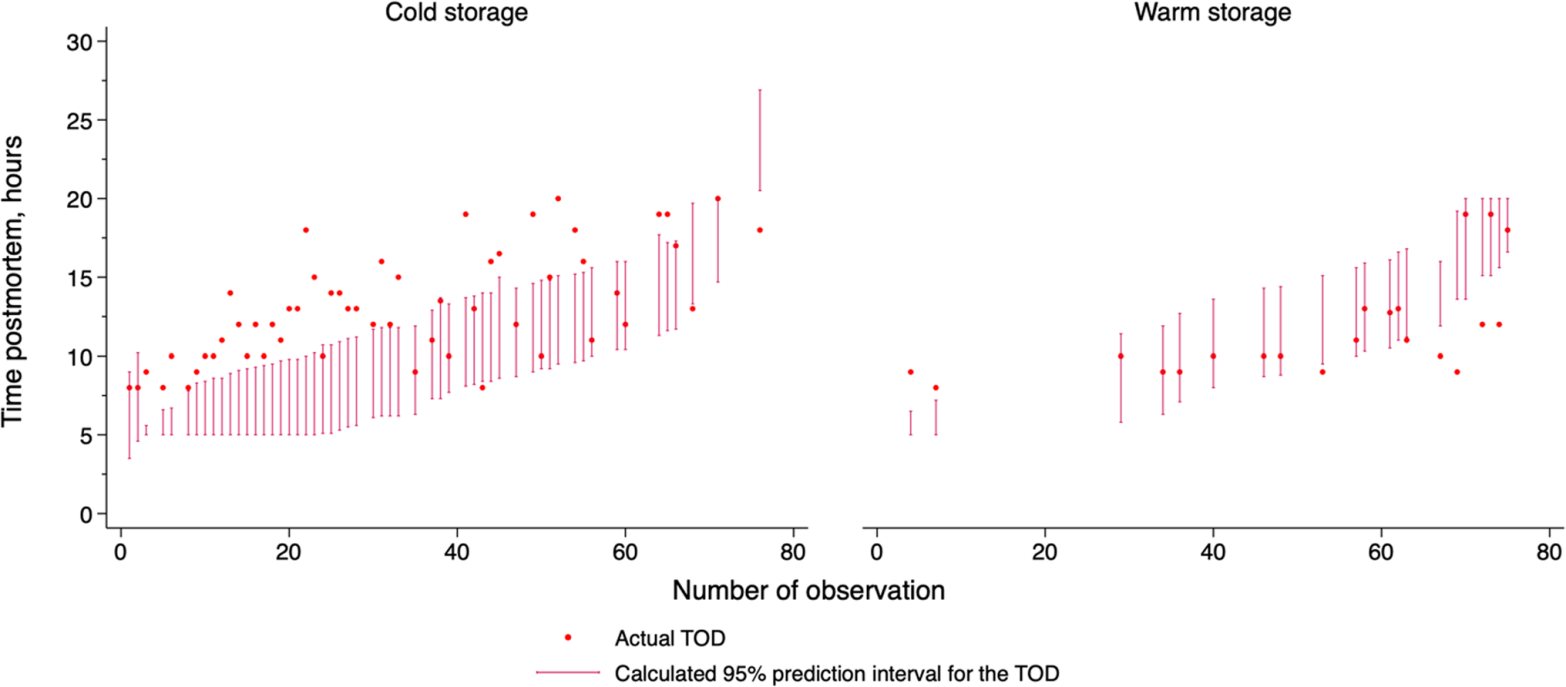
Actual TOD and the 95% prediction interval for the TSD are stratified by the storage mode. Abbreviations: TOD, time of death

Separate consideration of the cases stored at warmer and colder ambient temperatures revealed a more distinct picture: the 21 cases stored at warmer temperatures showed agreement of the actual TOD with the calculated 95% prediction interval for the TSD in 13/21 cases (61.9% [95% CI: 38.4 to 81.9]), whereas in the 55 cases stored at colder temperatures there was agreement in only 15/55 cases (27.3% [95% CI: 16.1 to 41.0]) of the cases. In cold stored corpses, among 95% prediction intervals not compatible with the actual TOD, 8% of the 95% prediction intervals overestimated the actual TOD (*n* = 3), and 93% underestimated the actual TOD (*n* = 37), whereas, in warm stored corpses, 75% of the 95% prediction intervals overestimated the actual TOD (*n* = 6) and 25% underestimated the actual TOD (*n* = 2; Fig. 1).

In the next step, we aim to examine the effect of (i) time from death until admission, (ii) time from admission until measurement, (iii) body mass index, (iv) body surface area, and (v) mode of storage on the odds of compatibility between the actual TOD and the 95% prediction interval for the TSD.

### Logistic regression

In univariable regression, with every hour increase in admission time, individuals had 0.76 times the odds of agreement in the actual TOD and the 95% prediction interval for the TSD (95% CI: 0.62 to 0.95). There was moderate evidence for a change in odds with every hour increase in admission time (*p* = 0.01). With every hour increase in storage time, individuals had 1.14 times the odds of agreement in the actual TOD and the 95% prediction interval for the TSD (95% CI: 0.97 to 1.34). No evidence for a change in odds with every hour increase in storage time was observed (*p* = 0.13). With every unit increase in BMI, individuals had 1.10 times the odds of agreement in the actual TOD and the 95% prediction interval for the TSD (95% CI: 1.02 to 1.20). There was moderate evidence for a change in odds with every unit increase in BMI (*p* = 0.02). With every 100 cm^2^ (1 dm^2^) increase in body surface area, individuals had 1.02 times the odds of agreement in the actual TOD and the 95% prediction interval for the TSD (95% CI: 1.00 to 1.04). There was moderate evidence for a change in odds with every 1 dm^2^ increase in body surface area (*p* = 0.04). Warm-stored individuals had 4.33 times the odds of agreement in the actual and 95% prediction interval for the TOD that cold-stored individuals had (95% CI: 1.50 to 12.53). There was moderate evidence for a change in odds when comparing warm- and cold-stored individuals (*p* = 0.01), with warm-stored individuals exhibiting increased odds of agreement in the actual TOD and 95% prediction interval for the TSD compared to cold-stored individuals. In contrast, no evidence for a change in the odds of agreement in the actual TOD and the 95% prediction interval for the TSD with every year increase in age (OR: 1.02 [95% CI: 0.98 to 1.05], *p* = 0.37), with every 1° C increase in rectal temperature (OR: 0.99 [95% CI: 0.87 to 1.15], *p* = 0.99), or when comparing male and female individuals (OR: 1.01 [95% CI: 0.39 to 2.63], *p* = 0.98) was found.

Next, we adjusted for important confounders of the association between the factors of interest and the odds of compatibility between the actual TOD and the 95% prediction interval for the TSD (Supplementary Figs. 1–4). When adjusting for storage mode, with every hour increase in admission time, individuals had 0.81 times the odds of agreement in the actual TOD and 95% prediction interval for the TSD (95% CI: 0.65 to 1.01). Weak evidence for a change in odds with every hour increase in admission time was observed when adjusting for the mode of storage (*p* = 0.06). With every hour increase in storage time, individuals had 1.14 times the odds of agreement in the actual TOD and 95% prediction interval for the TSD (95% CI: 0.97 to 1.34). No evidence for a change in odds with every hour increase in storage time was observed (*p* = 0.13). When adjusting for age, with every unit increase in body mass index, individuals had 1.11 times the odds of agreement in the actual TOD and 95% prediction interval for the TSDs (95% CI: 1.02 to 1.21). There was moderate evidence for a change in odds with every unit increase in body mass index when adjusting for confounder variables (*p* = 0.02). When adjusting for age, with every 1 dm^2^ increase in body surface area, individuals had 1.03 times the odds of agreement in the actual TOD and 95% prediction interval for the TSDs (95% CI: 1.004 to 1.05). There was moderate evidence for a change in odds with every unit increase in body mass index when adjusting for confounder variables (*p* = 0.02). When adjusting for age and admission time, warm-stored individuals had 4.40 times the odds of agreement in the actual TOD and 95% prediction interval for the TSD that cold-stored individuals had (95% CI: 1.24 to 15.65). There was moderate evidence for a change in odds when comparing warm and cold-stored individuals and adjusting for confounding variables (*p* = 0.02). No evidence of linear predictor misspecification or potentially influential values was observed.

When exploring an interaction term between admission time and mode of storage, there was moderate evidence for this interaction (*p* = 0.01; Univariable Wald test for the interaction term between time from death until admission and the mode of storage). When adjusting for the storage mode, with every hour increase in admission time, cold-stored individuals had 0.55 times the odds of agreement in the actual TOD and 95% prediction interval for the TSD (95% CI: 0.36 to 0.83). There was moderate evidence for a change in odds with every hour increase in admission time in cold-stored individuals (*p* = 0.01). In contrast, when adjusting for the mode of storage, with every hour increase in admission time, warm-stored individuals had 1.38 times the odds of agreement in the actual and 95% prediction interval for the TSD (95% CI: 0.85 to 2.24). No evidence for a change in odds with every hour increase in admission time in warm-stored individuals was observed (*p* = 0.20) Table 2.

**Table 2:** Univariable and multivariable logistic regression to examine the effects of (i) time from death until admission, (ii) time from admission until measurement, (iii) body mass index, (iv) body surface area, and (v) mode of storage on the odds of agreement in the actual TOD and 95% prediction interval for the TSD

	Crude OR (95% CI)	P-value	Adjusted OR (95% CI)	P-value
Admission time^1^, hours	0.76 (0.62 to 0.95)	0.01	0.81 (0.65 to 1.01)	0.06
Storage time^2^, hours	1.14 (0.97 to 1.34)	0.13	1.14 (0.97 to 1.34)	0.13
BMI^3^, kg/m^2^	1.10 (1.02 to 1.20)	0.02	1.11 (1.02 to 1.21)	0.02
BSA^4^, dm^2^	1.02 (1.001 to 1.04)	0.04	1.03 (1.004 to 1.05)	0.02
Mode of storage^5^ (Ref: cold-storage)	4.33 (1.50 to 12.53)	0.01	4.40 (1.24 to 15.65)	0.02
**When allowing for an interaction between admission time and mode of storage**^**6**^				
Admission time in corpses warm-stored^1^, hours	1.06 (0.78 to 1.46)	0.70	1.38 (0.85 to 2.24)	0.20
Admission time in corpses cold-stored^1^, hours	0.70 (0.54 to 0.91)	0.01	0.55 (0.36 to 0.83)	0.01

^1^Adjusted for mode of storage. ^2^No adjustment. ^3,4^Adjusted for age. ^5^Adjusted for cubic age and admission time. ^6^Univariable Wald test for an interaction term between time from death until admission and storage mode (*p* = 0.01). Abbreviations: *BMI* body mass index, *BSA* body surface area, *OR* odds ratio; 95% CI, 95% confidence interval

**Fig. 2:**
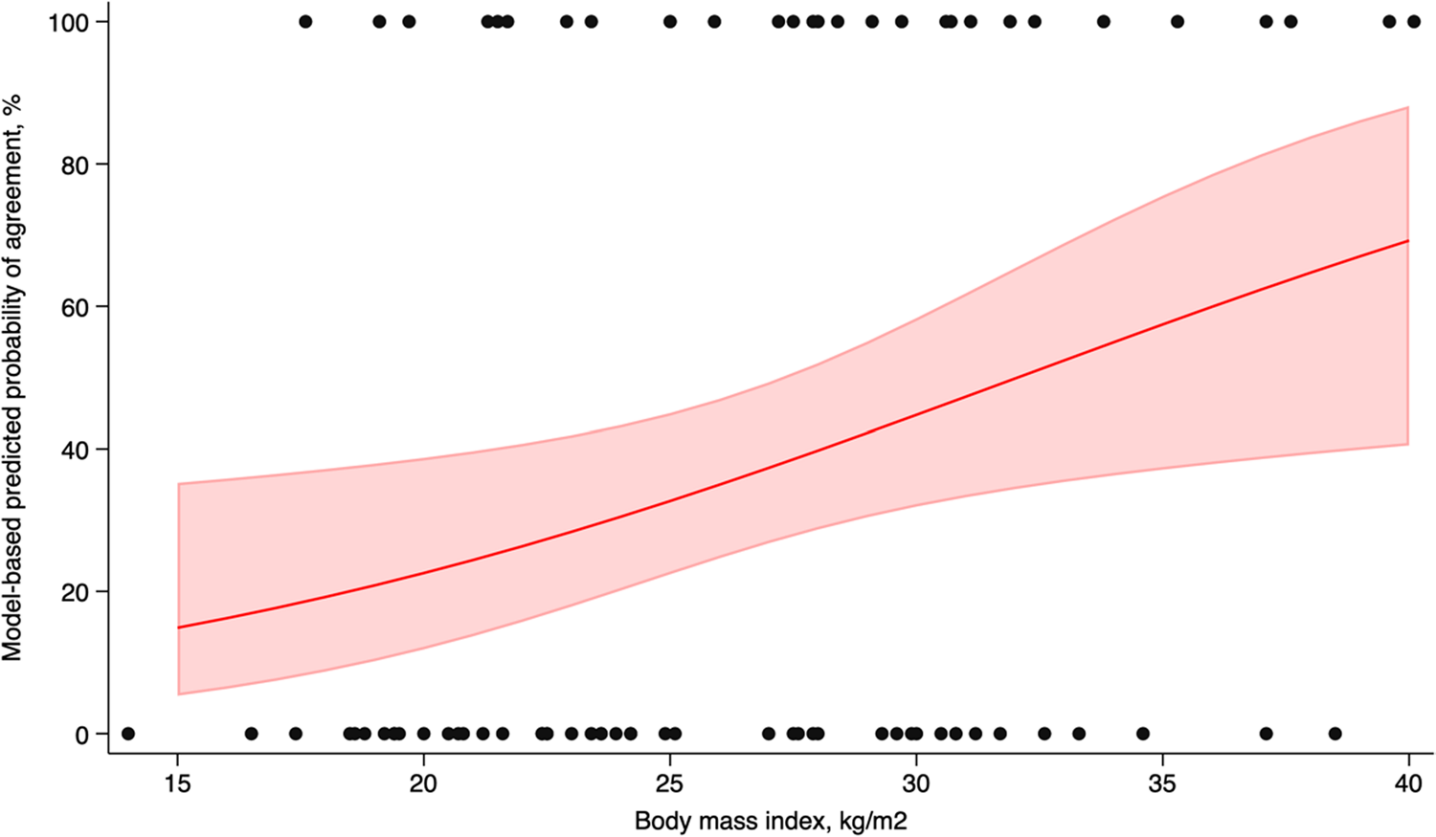
Model-based probabilities and 95% confidence interval of agreement in the actual and 95% prediction interval for the TSD are shown by the body mass. Scatter plots illustrate individual-level data of agreement according to the body mass index

Thus, the average individual (aged 70 years) with a BMI of 15 kg/m^2^ in this cohort had a 14.9% probability (95% CI: 5.3 to 35.2) of agreement in the actual and 95% prediction interval for the TSD. In comparison, individuals with a BMI of 35 kg/m^2^ had a 57.4% probability (95% CI: 37.1 to 75.6) of agreement in the actual and 95% prediction interval for the TSD (Fig. 2). The average individual (aged 70 years) with a BSA of 1.50 m^2^ in this cohort had a 16.0% probability (95% CI: 6.4 to 36.2) of agreement in the actual and 95% prediction interval for the TSD. In comparison, individuals with a BSA of 2.40 m^2^ had a 67.8% probability (95% CI:40.2 to 86.7) of agreement in the actual and 95% prediction interval for the TSD.

**Fig. 3:**
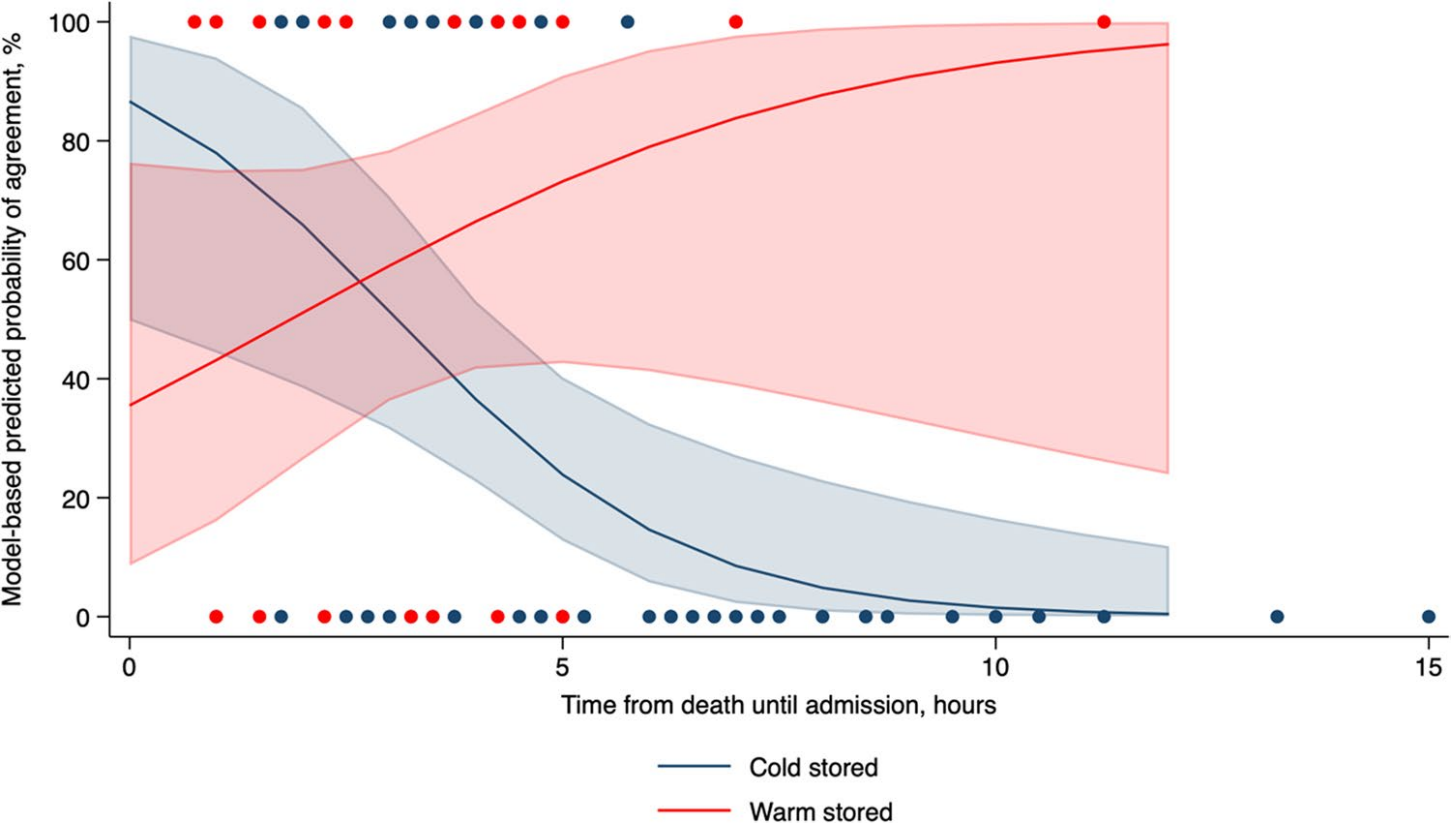
Model-based probabilities and 95% confidence interval of agreement in the actual and 95% prediction interval for the TSD are shown by admission time in warm-stored (red) and cold-stored (blue) individuals. Scatter plots illustrate individual-level agreement data according to the time from death until admission

When allowing for additional interaction between admission time and mode of storage, this meant that the average cold-stored individual in this cohort had a 51.3% probability (95% CI: 31.5 to 70.7) of agreement in the actual TOD and 95% prediction interval for the TSD when the time from death until admission was modelled 3 h, while the average cold-stored individual with times from death until admission of 7 h had a 8.5% probability (95% CI: 2.3 to 27.2) of agreement in the actual TOD and 95% prediction interval for the TSD. In contrast, the average warm-stored individual in this cohort had a 59.0% probability (95% CI: 36.3 to 78.4) of agreement in the actual TOD and a 95% prediction interval for the TSD when the time from death until admission was modelled 3 h. In comparison, the average warm-stored individual with times from death until the admission of 7 h had an 83.8% probability (95% CI: 38.8 to 97.7) of agreement in the actual TOD and a 95% prediction interval for the TSD (Fig. 3).

Having discovered that there is still a link between the time from death until admission and the odds of agreement between the actual TOD and the 95% prediction interval for the TSD, even after adjustment according to Henssge, especially in corpses that have been stored in cold temperatures, we aimed to explore the impact of accounting for potentially influential factors during this interval, such as varying ambient temperatures.

### (b) Taking a change in ambient temperature from death until measurement into account, assuming a plausible range of values for the ambient temperatures before admission – only temperature-based method

In this analysis, one case had to be excluded from the calculations at assumed ambient temperatures of 26 °C to 29 °C because the body and ambient temperatures were too close to each other to determine the TSD interval.

In the overall cohort, investigating a plausible range of ambient temperatures from death until admission, ranging from 19 °C to 29 °C (see calculation b), resulted in a marked increase in the agreement of the actual TOD with the 95% prediction interval for the TSD in this cohort.

The highest agreement in the actual TOD and 95% prediction interval for the TSD in our overall cohort was found at an assumed ambient temperature of 25 °C from death until admission with 59.2% (95% CI: 47.3 to 70.4; Table 3). When stratifying the cohort by the storage mode, a more pronounced improvement in the agreement in the actual TOD and 95% prediction intervals for the TSD was observed in the cold-stored cases. Here, the agreement in the actual TOD and 95% prediction intervals for the TSD improved with increasing assumed ambient temperature. The highest agreement in the actual TOD and 95% prediction intervals for the TSD in this cohort of cold-stored corpses was found at an assumed ambient temperature of 25 °C (60.0% [95% CI: 45.9 to 73.0]), without further improvement at higher temperatures. For warm-stored corpses, the agreement in the actual TOD and 95% prediction intervals for the TSD deteriorated with increasing assumed ambient temperatures (Supplementary Table 1).

**Table 3:** Percentages of agreement in actual TOD and 95% prediction interval for the TSD when assuming a plausible range of ambient temperatures (26 °C to 29 °C) from death until admission to the ILM. Temperature-based methods were used only (*n* = 75)

Assumed ambient temperature, °C	TOD above the 95% PI, % (n)	TOD compatible with 95% PI, % (n)	TOD below the 95% PI, % (n)
Storage temperature^1^	51.32 (39)	36.84 (28)	11.84 (9)
19	39.47 (30)	48.58 (37)	11.84 (9)
20	36.84 (28)	50.00 (38)	13.16 (10)
21	34.21 (26)	52,63 (40)	13.16 (10)
22	38.89 (25)	50.00 (38)	17.11 (13)
23	31.58 (24)	51.32 (39)	17.11 (13)
24	27.63 (21)	55.26 (42)	17.11 (13)
25	23.68 (18)	59.21 (45)	17.11 (13)
26	22.67 (17)	56.00 (42)	21.33 (16)
27	21.33 (16)	57.33 (43)	21.33 (16)
28	18.67 (14)	56.00 (42)	25.33 (19)
29	18.67 (14)	56.00 (42)	25.33 (19)

^1^According to the calculation in a), constant temperatures were assumed from death until measurement. Abbreviations: TOD, time of death; PI, prediction interval

After an exploratory approach assuming a plausible range of ambient temperatures, we aim to confirm these cohort-specific findings using the compound method.

### (c) Taking a plausible range of ambient temperatures from death until admission into account – compound method

When the 95% prediction interval for the TSD was estimated using a cohort-specific ambient temperature of 25 °C (as derived for this cohort in calculation b) but using both temperature-based and non-temperature-based methods, agreement in the actual TOD and 95% prediction interval for the TSD was observed in 60.5% (95% CI: 48.6 to 71.6). In cold-stored corpses, agreement in the actual TOD and 95% prediction interval for the TSD was observed in 61.8% (95% CI: 47.7 to 74.6), and in warm-stored corpses, agreement was observed in 57.1% (95% CI: 34.0 to 78.1).

### Non-temperature-based methods

Non-temperature-based methods complement temperature-based TSD diagnostics. When non-temperature-based diagnostics are positive and the upper or lower time limit is within the 95% prediction interval for the TOD, non-temperature-based methods can further narrow the 95% prediction interval for the TSD. In the present cohort, non-temperature-based methods limited the 95% prediction interval for the TSD in 38% of cases (*n* = 29) when the compound method was applied. In none of those cases did non-temperature-based truncation of the 95% prediction interval cause incompatibility between the actual TOD and the 95% prediction interval for the TOD. However, where non-temperature-based methods suggest a wider 95% prediction interval, improved compatibility between the actual TOD and the 95% prediction interval for the TSD was observed in three cases.

## Discussion

Here, we aim to externally validate the temperature-based and compound methods by Henssge in 76 cases with a known TOD. Moreover, we strive to investigate the effect of (i) time from death until admission, (ii) time from admission until measurement, (iii) body mass index, (iv) body surface area, and (v) mode of storage on the odds of agreement in the actual TOD and 95% prediction intervals for the TSDs to understand residual associations of those interest factors with the compatibility between the actual TOD and the 95% prediction interval for the TSD after adjustment for the factors incorporated in the Henssge equations [8]. In summary, we found an overall low to moderate agreement of the compound method described by Henssge in this cohort. The actual TOD and 95% prediction interval for the TSD were compatible in only 36.8% of the cases (95% CI: 26.1 to 48.7) but in 61.9% of the warm-stored cases (95% CI: 38.4 to 81.9).

When examining the effect of (i) admission time, (ii) storage time, (iii) body mass index, (iv) body surface area, and (v) mode of storage on the odds of agreement in the actual TOD and 95% prediction interval for the TSD, we found weak evidence for a 0.81 (95% CI: 0.65 to 1.01) decrease in odds with every hour increase in admission time (*p* = 0.06) when adjusting for important confounders. When allowing for interaction with storage mode, moderate evidence for a 0.55 (95% CI: 0.36 to 0.83) decrease in odds with every hour increase in admission time was observed in cold-stored corpses (*p* = 0.01). No evidence for a change in odds with every hour increase in admission time was observed in warm-stored corpses (OR: 1.38 [95% CI: 0.85 to 2.24], *p* = 0.20). The study was likely underpowered to detect any effects because of the smaller sample size in the warm-stored cohort and the wide-ranging 95% confidence interval. In line, in warm-stored corpses, the actual TOD and 95% prediction interval for the TSD were compatible in 61.9% (95% CI: 38.4 to 81.9), and in cold-stored corpses, the actual TOD and 95% prediction interval for the TSD were compatible in 27.3% (95% CI: 16.1 to 41.0). Notably, 95% prediction intervals obtained from the Henssge equations tended to underestimate the actual TOD in cold-stored corpses while they tended to overestimate the actual TOD in warm-stored corpses. This suggests that the model was developed based on a group of corpses stored under ambient temperatures between warm and cold-stored corpses here. A range of ambient temperatures between 9.2 and 17.4 °C is reported in the Henssge publication [8], without a population mean reported. Our findings suggest that the Henssge model does not accurately account for variations in ambient temperatures.

Henssge neglected an interval from death until admission of up to six hours when establishing the model on 39 corpses and extrapolated ambient temperatures under storage conditions to the interval from death until admission [8]. While this assumption may have been valid for most cases on which the model was established—consisting of corpses with a known time of death, which were most likely kept at room temperature—it is not necessarily valid for all cases of the cohort or cases of other cohorts. As expected, extrapolation of ambient temperatures seemed less plausible in cold-stored corpses. This finding is supported by moderate evidence for a 4.40 (95% CI: 1.24 to 15.65) increase in odds when comparing warm and cold-stored corpses. While no evidence for a change in odds with every hour increase in admission time was found in the overall cohort, moderate evidence for a change in odds with every hour increase in admission time was found in cold-stored corpses when allowing for an interaction between admission time and storage mode. Notably, model-based predicted probabilities of agreement in the actual TOD and 95% prediction intervals for the TSD for the average cold-stored individual with an admission time of modelled 3 h was 51.3% (95% CI: 31.5 to 70.7), and for the average individual with an admission time of 7 h was 8.5% (95% CI: 2.3 to 27.2). As the time between death and admission increases, and thus the degree of extrapolation, agreement in actual TOD and the 95% prediction interval deteriorates in cold-stored corpses. This demonstration is mainly for illustrative purposes of potential problems when neglecting the time from death until admission and extrapolating storage temperatures for the time from death until admission, but it also calls for methods to account for changes in ambient temperatures at the death scene [21]. Generally, it must be noted that without external validation, the Henssge equations are prone to overfitting and limited transportability.

Although body weight is accounted for in the Henssge model, the impact of other characteristics likely influences cooling kinetics. For instance, factors such as body surface or insulation comprising body fat composition were not considered in the Henssge equations yet are likely to impact cooling kinetics. We found moderate evidence for a 1.11 (95% CI: 1.02 to 1.21) increase in odds with every unit increase in BMI after adjustment for important confounders. This 11% increase in the odds of agreement in actual TOD and a 95% prediction interval for the TSD with every unit increase in BMI implies a residual association of the BMI with the correct estimation of the 95% prediction interval after adjustment for weight in the Henssge model. It is noticeable that the model predicted probability of agreement in the actual and 95% prediction interval for the TSD for the average individual with a BMI of 15 kg/m^2^ was 14.9% (95% CI 5.3 to 35.2) and for the average individual with a BMI of 35 kg/m^2^ was 57.4 (95% CI: 37.1 to 75.6) when adjusting for confounding variables.

Moreover, we found moderate evidence for a 1.03 (95% CI: 1.004 to 1.05) increase in odds with every unit increase in BSA after adjustment for important confounders. Again, this implies an association of the BSA with the correct estimation of the 95% prediction interval after adjusting for weight in the Henssge model. The model predicted probability of agreement in the actual and 95% prediction interval for the TSD for the average individual with a BSA of 1.5 m^2^ was 16.0% (95% CI: 6.4 to 36.2) and for the average individual with a BSA of 2.40 m^2^ was 67.8 (95% CI:40.2 to 86.7) when adjusting for confounding variables.

### Comparison with other studies

Although the compound method described by Henssge is widely used internationally, only a few studies have aimed to validate the model externally. Most studies that aim to validate the model externally report a good agreement in TSD intervals delimited by police investigations and the 95% prediction interval for the TSD. The agreement, defined as the complete overlap of both intervals, ranges between 63.6% and 89.1% in those studies [14–16]. None of these studies reported the predictive ability of the Henssge model in a cohort with a known TOD. Other studies included cases based on a known TOD reported a poor fit of the Henssge model with an agreement in the actual TOD and the 95% prediction interval for the TSD in 43% and 50% [12, 17]. However, one of the latter studies had a small sample size of *n* = 8 [17]. A study on 12 deceased with a known actual TOD reported good agreement in the actual TOD and the 95% prediction interval for the TOD in 11 of these cases yet did not consider the statistical uncertainty in the point estimate (91.7% [95% CI: 61.5 to 99.8^1^]) [18]. The results of another study on 30 deceased individuals are challenging to interpret regarding the agreement between the actual TOD and the 95% prediction interval for the TSD. This is because deviations are only reported as average hours across aggregated groups of cases [19].

Notably, most studies that investigated the Henssge model and reported a good predictive ability compared 95% prediction intervals for the TSD and TSD intervals delimited by police investigations.

In a multicenter study on 76 cases [14], an overall agreement of 80.3%, defined based on the complete overlap between the 95% prediction interval for the TSD and a TSD interval delimited by police investigations interval, was reported. The authors divided the overall cohort into two case groups: Group 1 included cases in which conditions at the death scene were known or could be narrowed down (*n* = 46). In this cohort, complete overlap between both intervals was observed in 41 cases (89% [95% CI: 83 to 98^2^]). Five cases showed a partial overlap in the 95% prediction interval for the TSD and the TSD interval delimited by police investigations (11% [95% CI: 4 to 20^3^]). Cohort 2 comprised cases where conditions at the death scene remained uncertain (*n* = 30). In this cohort, complete overlap between both intervals was observed in 20 cases (67% [95% CI: 53 to 85^3^]). The remaining cases showed partial overlap in five cases (17% [95% CI: 3 to 35^3^]) and five with no overlap between the intervals (17% [95% CI: 2 to 35^3^]). Additionally, there was no clear definition of group allocation to both groups across the different centres. Furthermore, the study had limitations due to untransparent criteria used to delimit the TSD interval by police investigations.

Two further studies also compared the overlap between the 95% prediction intervals for the TSD and a TSD interval delimited by police investigations. These studies were, at least in part, based on the same cases [15, 16]. Most of the examinations were conducted at the site of discovery. In the first study, only the temperature-based methods were used [15]. Of the 60 cases examined, 50 showed a complete overlap between the time intervals (83% [95% CI: 75 to 92^3^]), and in 10 cases, there was a partial overlap of intervals (16.7% [95% CI: 8 to 26^3^]). In the second study [16], 95% prediction intervals for the TSD, according to Henssge, were compared with TSD intervals delimited by police investigations in 44 cases. Again, cases were divided into two groups: Group 1 included 35 cases in which the TSD intervals delimited by police investigations could be narrowed down to less than 4 h. In this group, 28 cases showed complete overlap of both intervals (80% [95% CI: 69 to 92^3^]). However, 6 cases showed only a partial overlap of both intervals (17% [95% CI: 6 to 29^3^]), and in one case, there was no overlap in the two intervals (3% [95% CI: 0 to 15^3^]). The latter case can be explained from today's point of view against the background of more recent results on the time limit of re-establishment of rigor mortis [4, 5]. Group 2 included cases with TSD intervals delimited by police investigations of more than 4 h. For cohort 2, no results were presented except for describing the results as not contradictory.

While the abovementioned studies show ambiguities regarding their methodology, a comparison between 95% prediction intervals for the TSD and a TSD interval delimited by police investigations generally only allows limited conclusions to be drawn as to whether the actual TOD and 95% prediction interval for the TSD agree. These studies rely on comparing the 95% prediction intervals for the TSD and a TSD interval delimited by police investigations and then defining them as completely, partially, or not overlapping at all. Notably, cases can be constructed where the 95% prediction interval for the TOD and the TSD intervals delimited by police investigations overlap, and still, the actual TOD is not compatible with the 95% prediction interval, particularly where the TSD interval delimited by police investigations is wider than the 95% prediction interval for the TOD. Noteworthy, the TSD interval delimited by police investigations are not statistical intervals but instead ranges of plausible values determined empirically. Therefore, conducting standard hypothesis testing on those intervals is not appropriate and may provide unreliable conclusions.

Two studies previously reported low sensitivity of the Henssge method. In an evaluation of 8 cases, overall, 50% agreement in the actual TOD and 95% prediction interval for the TSD was found (50% [95% CI: 16 to 84^3^]) [17]. In a more recent publication of a standardised experimental study, the authors reported that they found the actual TOD compatible with the 95% prediction interval for the TSD in 36 of 84 cases (42.9% [95% CI: 32 to 54^4^]] and incompatible with the 95% prediction interval for the TSD in 48 of 84 cases (57.1% [95% CI: 46 to 68^4^]) [12]. In addition, they reported a trend towards systematic overestimation of the time since death, especially in cases with a large body mass and surface. We systematically overestimated the 95% prediction interval for the TSD in warm-stored corpses. We found an association with body mass index; however, in this cohort, we observed an inverse association where corpses with higher BMIs had increased odds of agreement.

### Exploratory approach

When assuming a plausible range of ambient temperatures during the neglected interval from death until admission (calculation b), improved agreement in the actual and 95% prediction interval for the TSD was observed in cold-stored but not warm-stored individuals when using temperature-based methods only. An assumed ambient temperature of 25 °C from death until admission led to the highest agreement in the actual TOD and 95% prediction interval for the TSD in cold-stored individuals (60.0% [95% CI: 45.9 to 73.0]). This makes perfect sense because, as discussed above, the assumption of cold ambient temperatures from death until admission for corpses stored at cold temperatures is likely a false assumption for this cohort. The obtained value from part (b) is cohort-specific and not transferable to other cohorts with a different baseline and medical characteristics composition. The fact that the assumed ambient temperature during that time yields above room temperature is plausible given that most corpses were likely stored at room temperature and the clothing and covering state of the deceased. Improved agreement in the actual and 95% prediction interval for the TSD in this cohort is confirmed using the compound method with an assumed ambient temperature of 25 °C from death until admission (60.5% [95% CI: 48.6 to 71.6]).

Consideration of a weighted average of ambient temperatures in cases with a change in ambient temperatures is not standardly proposed in forensic death time diagnostics, as examinations should be performed at the site of discovery, and the model was developed for application to cases with constant ambient temperature, clothing and covering state [1]. However, using a weighted-average ambient temperature was suggested for practical casework where changes in ambient temperatures occurred [1, 22]. Similarly, a weighted-average ambient temperature use was suggested for accumulated degree days in cases with long intervals between death and measurement and putrefactive changes [23]. While using this weighted-average ambient temperature might improve the predictive ability in cases where a change in ambient temperature is a plausible assumption, it must be noted that constant cooling kinetics are assumed when using the model according to Henssge. Thus, the use of weighted-average ambient temperatures can only be considered a crude approximation. Again, the plausible ambient temperature of 25 °C used here is a cohort-specific value that cannot be transferred to other cohorts.

We found a low to intermediate agreement in the actual TOD and the 95% prediction interval for the TSD in the overall and warm-stored cohort. Residual associations of BMI and BSA with the odds of agreement in the actual TOD and 95% prediction intervals for the TSD suggest that incorporating BMI or BSA into the model could improve its predictive ability. Systematic overestimation of the 95% prediction interval for the TSD in warm-stored corpses and underestimation in cold-stored corpses suggests that this component is missing from the original equation. It is essential to exercise caution when using temperature-based death time estimation on individual cases. Using an exclusionary approach to evaluate alibis or competing investigation results is not justified based on current data.

The strengths of the present work are its blinded study design, its comparison of 95% prediction intervals according to Henssge to an actual TOD and the integration of temperature-based and non-temperature-based methods. The design of the present study is deliberately based on Henssge’s studies to ensure comparability [8–11, 13]. Limitations include uncertainty in ambient temperature and environmental conditions at the place of death and the interval from death until admission to the Institute of Legal Medicine. Due to the experimental approach, observed agreement in the actual TOD and 95% prediction interval for the TSD might not be directly transportable to cases at the crime scene, with factors such as substantial internal or external injury with relevant blood loss likely present. Moreover, changes in the ambient temperature are frequently present at the crime scene. These factors might further impair the predictive ability of the Henssge method.

Approaches to improve the estimation of the actual TOD should include a review and revision of currently used methods on a large number of corpses with clearly defined inclusion and exclusion criteria. External validation is crucial for prediction modelling; future studies must carefully choose datasets on which they want to validate their model externally. Furthermore, future models must consider and deal with the left truncation of the data collected. They should include and assess bodies with specific characteristics that are known to influence cooling kinetics, such as hypothermia, hyperthermia [1, 12], and significant blood loss, to make the model more widely applicable.

## Supplementary Information

Below is the link to the electronic supplementary material.

Supplementary file1 (PDF 363 KB)

## Data Availability

The datasets generated during and/or analysed during the current study are available from the corresponding author on reasonable request.

## Footnotes

1 A binomial exact 95% confidence interval was calculated by the authors of this study.

2 95% confidence intervals for multinomial proportions were calculated by the authors of this study.

3 A binomial exact 95% confidence interval was calculated by the authors of this study.
