# Supplementary material for: An assessment of the Henssge method for forensic death time estimation in the early post-mortem interval
Source: Int J Legal Med. 2024 Sep 24;139(1):105–17. doi: 10.1007/s00414-024-03338-5 (PMC11732859; doi:10.1007/s00414-024-03338-5)
Supplement: Supplementary file 1 — Supplementary file1 (PDF 363 KB) [file 414_2024_3338_MOESM1_ESM.pdf]

Supplementary materials

An assessment of the Henssge method for forensic death time estimation in the early post-mortem interval

International Journal of Legal Medicine

Fabian Heinrich[1,2,3*], Felix Rimkus-Ebeling[1*], Eric Dietz[1], Tobias Raupach[4], Benjamin Ondruschka[1], Sven Anders-Lohner[1]

[1]Institute of Legal Medicine, University Medical Center Hamburg-Eppendorf, Hamburg, Germany

[2]Department of Medical Statistics, London School of Hygiene and Tropical Medicine, London, UK

[3]Centre for Data and Statistical Science for Health, London School of Hygiene and Tropical Medicine, London, UK

[4]Institute for Medical Education, University Hospital Bonn, Bonn, Germany

*Shared first authorship.

Corresponding author:

Prof. Dr. med. Sven Anders-Lohner, MME

University Medical Center Hamburg-Eppendorf

Institute of Legal Medicine

Butenfeld 34

22529 Hamburg

Germany

s.anders-lohner@uke.de

Table of contents

- **Supplementary Figure 1.** Directed acyclic graph for the association between the time from death until admission and the agreement in the actual and 95% prediction interval for the TSD. Green illustrates the exposure of interest. Red illustrates clinically defined confounders. Blue illustrates the outcome of interest.
- **Supplementary Figure 2.** Directed acyclic graph for the association between the time from admission until measurement and the agreement in the actual and 95% prediction interval for the TSD. Green illustrates the exposure of interest. Red illustrates clinically defined confounders. Blue illustrates the outcome of interest.
- **Supplementary Figure 3.** Directed acyclic graph for the association between the body mass index and the agreement in the actual and 95% prediction interval for the TSD. Green illustrates the exposure of interest. Red illustrates clinically defined confounders. Blue illustrates the outcome of interest.
- **Supplementary Figures 4.** Directed acyclic graph for the association between the body surface area and the agreement in the actual and 95% prediction interval for the TSD. Green illustrates the exposure of interest. Red illustrates clinically defined confounders. Blue illustrates the outcome of interest.
- **Supplementary Figure 5.** Directed acyclic graph for the association between the mode of storage and the agreement in the actual and 95% prediction interval for the TSD. Green illustrates the exposure of interest. Red illustrates clinically defined confounders. Blue illustrates the outcome of interest.
- **Supplementary Table 1.** Percentages of agreement in actual TOD and 95% prediction interval for the TSD when assuming a plausible range of ambient temperatures (26°C to 29°C) for the interval from death until admission to the ILM stratified by the mode of storage. Temperature-based methods were used only (n=76).

Supplementary Figures 1. Directed acyclic graph for the association between the time from death until admission and the agreement in the actual and 95% prediction interval for the TSD. Green illustrates the exposure of interest. Red illustrates clinically defined confounders. Blue illustrates the outcome of interest.

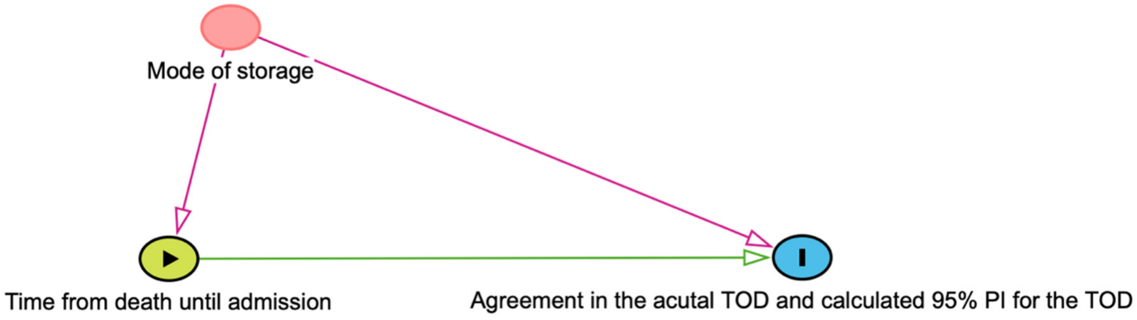

Supplementary Figures 2. Directed acyclic graph for the association between the time from admission until measurement and the agreement in the actual and 95% prediction interval for the TSD. Green illustrates the exposure of interest. Red illustrates clinically defined confounders. Blue illustrates the outcome of interest.

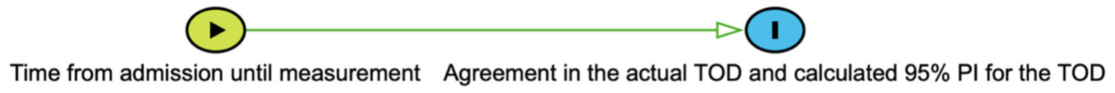

Supplementary Figures 3. Directed acyclic graph for the association between the body mass index and the agreement in the actual and 95% prediction interval for the TSD. Green illustrates the exposure of interest. Red illustrates clinically defined confounders. Blue illustrates the outcome of interest.

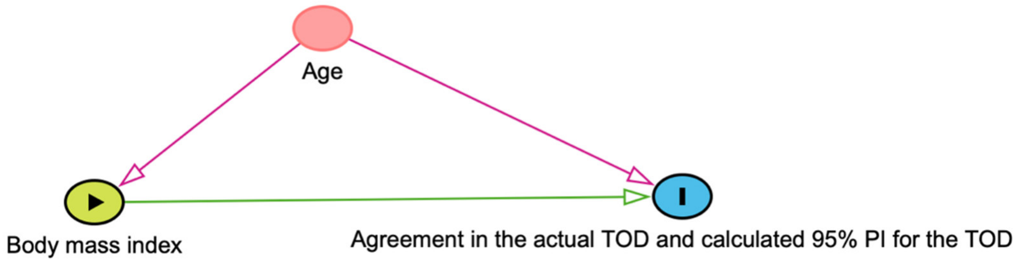

Supplementary Figures 4. Directed acyclic graph for the association between the body surface area and the agreement in the actual and 95% prediction interval for the TSD. Green illustrates the exposure of interest. Red illustrates clinically defined confounders. Blue illustrates the outcome of interest.

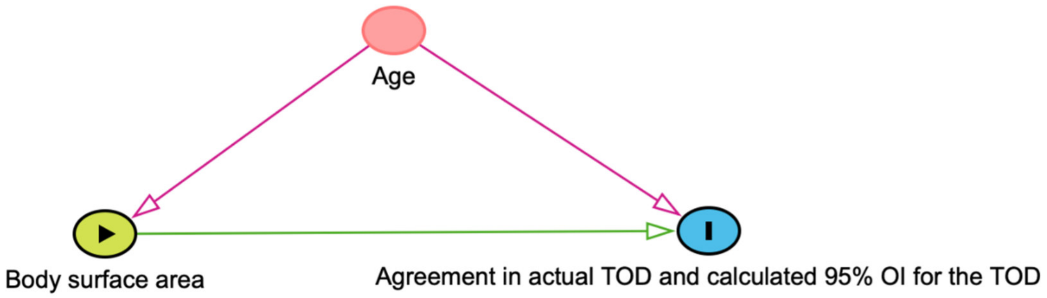

Supplementary Figures 5. Directed acyclic graph for the association between the mode of storage and the agreement in the actual and 95% prediction interval for the TSD. Green illustrates the exposure of interest. Red illustrates clinically defined confounders. Blue illustrates the outcome of interest.

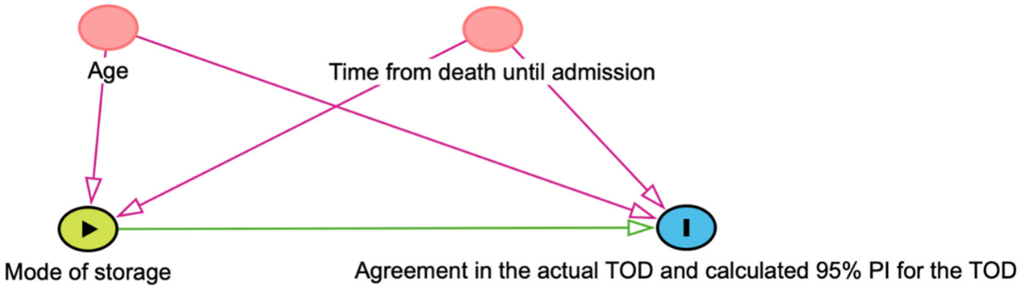

Supplementary Table 1. Percentages of agreement in actual TOD and 95% prediction interval for the TSD when assuming a plausible range of ambient temperatures (26°C to 29°C) for the interval from death until admission to the ILM stratified by the mode of storage. Temperature-based methods were used only (n=76).

	**Cold-stored corpses** Mean storage temperature: 9.29°C			**Warm-stored corpses** Mean storage temperature: 19.68°C		
**Assumed ambient temperature**, °C	**TOD above the 95% PI**, n (%)	**TOD compatible with 95% PI**, n (%)	**TOD below the 95% PI**, n (%)	**TOD above the 95% PI**, n (%)	**TOD compatible with 95% PI**, n (%)	**TOD below the 95% PI**, n (%)
	N=55			N=21		
Storage temperature[1]	67.27 (37)	27.27 (15)	5.45 (3)	9.52 (2)	61.9 (13)	28.57 (6)
19	50.91 (28)	41.82 (23)	7.27 (4)	9.52 (2)	66.67 (14)	23.81 (5)
20	47.27 (26)	45.45 (25)	7.27 (4)	9.52 (2)	61.90 (13)	28.57 (6)
21	43.64 (24)	49.09 (27)	7.27 (4)	9.52 (2)	61.90 (13)	33.33 (7)
22	41.82 (23)	47.27 (26)	10.91 (6)	9.52 (2)	57.14 (12)	33.33 (7)
23	40.00 (22)	49.09 (27)	10.91 (6)	9.52 (2)	57.14 (12)	33.33 (7)
24	34.55 (19)	54.55 (30)	10.91 (6)	9.52 (2)	57.14 (12)	33.33 (7)
25	29.09 (16)	60.00 (33)	10.91 (6)	9.52 (2)	57.14 (12)	33.33 (7)
26	27.27 (15)	60.00 (33)	12.73 (7)	10.00 (2)	45.00 (9)	45.00 (9)
27	25.45 (14)	60.00 (33)	14.55 (8)	10.00 (2)	50.00 (10)	40.00 (8)
28	23.64 (13)	60.00 (33)	16.36 (9)	5.00 (1)	45.00 (9)	50.00 (10)
29	21.82 (12)	60.00 (33)	18.18 (10)	10.00 (2)	45.00 (9)	45.00 (9)

[1]According to calculation a), constant temperatures were assumed from death until measurement.
Abbreviations: TOD, time of death; PI, prediction interval.

One case had to be excluded from calculations at assumed ambient temperatures of 26°C to 29°C because the body and ambient temperatures were too close to each other to allow determination of the TSD interval.
